# Experiences of Performing Daily Activities in Middle-Aged and Older Autistic Adults: A Qualitative Study

**DOI:** 10.1007/s10803-022-05473-7

**Published:** 2022-02-25

**Authors:** Ye In Jane Hwang, Kitty-Rose Foley, Kieran Elley, Scott Brown, Dawn Joy-Leong, Xue Li, Rachel Grove, Julian Trollor, Elizabeth Pellicano, Lidan Zheng

**Affiliations:** 1 UNSW Ageing Futures Institute, UNSW Sydney, Sydney, NSW Australia; 2 School of Population Health, UNSW Sydney, Level 2 Samuels Building, Sydney, NSW 2052 Australia; 3 Faculty of Health, Southern Cross University, Bilinga, QLD Australia; 4 Faculty of Health, University of Technology Sydney, Ultimo, NSW Australia; 5 Creative Robotics Lab, UNSW Art & Design, Paddington, NSW Australia; 6 UNSW Art & Design, Paddington, NSW Australia; 7 School of Population Health, UNSW Centre for Primary Health Care and Equity, Sydney, NSW Australia; 8 School of Education, Faculty of Arts, Macquarie University, Sydney, NSW Australia; 9 Department of Developmental Disability Neuropsychiatry, School of Psychiatry, UNSW Medicine & Health, Sydney, NSW Australia; 10 Neuroscience Research Australia, Randwick, NSW Australia

**Keywords:** Adulthood, Ageing, Daily living, Person-environment fit, Independence

## Abstract

This is the first study to investigate instrumental activities of daily living in older autistic adults. We conducted interviews with fifteen adults (mean age = 60.1, SD = 7.4, range = 50–73) from Australia with no intellectual disability. Analysis included both deductive and inductive steps, to categorise responses using the Occupational Performance Model Australia and identify themes across participants’ experiences. Strengths and challenges were unique to the individual, as were the methods they had developed to manage tasks. Challenges occurred mostly at the interaction between aspects of the environment (sensory, cognitive, social and cultural) and personal factors such as health conditions and sensory sensitivities. Enhanced person-environment fit is needed, as is a shift in wider sociocultural attitudes to enable comfort and autonomy in later life.

**Supplementary Information:**

The online version contains supplementary material available at 10.1007/s10803-022-05473-7.

## Introduction

Recently, increased attention has been given to autistic people in middle-to-late adulthood (Sonido et al., 2020), with numerous commentaries (Bennett, 2016; Wright et al., 2016), expert consensus statements (Edelson et al., 2021; Roestorf et al., 2019) and reviews (Amanullah et al., 2020; Ruggieri et al., 2019) calling for more research and funding to better understand the health and wellbeing of this population. Despite such attention, there remain important gaps in our understanding regarding the daily life and experiences of autistic adults as they enter later life.

As people age, their support needs change. Consequently, there is a large body of research dedicated to understanding and designing supports to increase independent living for older adults from the general population. Activities of daily living (ADLs) are a set of essential and routine tasks that are fundamental to independent care of oneself (e.g., bathing, dressing). Instrumental activities of daily living (IADLs) refer to more complex tasks that are important for independent living, such as grocery shopping or managing finances (Lawton & Brody, 1969; Spector et al., 1987). Both ADLs and IADLs have received considerable attention in the community-dwelling elderly. They are commonly used as outcome measures of support needs and independent living in older adults, with a systematic review finding 140 relevant papers describing 50 different measures of ADLs (Hopman-Rock et al., 2019). Competence in these domains is positively associated with quality of life (Gobbens, 2018; Lyu & Wolinsky, 2017). A range of occupational therapy interventions seek to improve IADL performance in community-dwelling non-autistic older adults, with promising evidence of their effectiveness (Hunter & Kearney, 2018).

A small number of studies have demonstrated low levels of independent living for adults on the autism spectrum of all ages, compared to non-autistic people (Howlin & Magiati, 2017). In a recent think tank entitled “Strategies for Research, Practice and Policy for Autism in Later Life”, autistic participants identified “maintaining an independent lifestyle” as a key concern as they age (Edelson et al., 2021, p. 387). However, only a handful of studies have specifically investigated daily activities for autistic adults. A longitudinal examination of ADLs over a 10-year period from adolescence to adulthood (N = 397, mean age at end of study = 31.23 years) found improvement in ADLs that eventually plateaued during the late 20s, with most not achieving independence in ADLs by this time (Smith et al., 2012). A cross-sectional evaluation including n = 72 adults over 40 (28% of sample) found only 40% of this group was independent across four ADLs: eating, dressing, bathing, and toileting (Fortuna et al., 2016). Half of the sample had an IQ less than 70 (50.2%), 5.1% of the sample had an IQ over 70, and for 44.7% of the sample, IQ was unknown. Another study observed ADLs and IADLs for a sample of autistic adults over 35 (N = 74, mean age = 49.9 years), 55% of whom also had moderate to severe intellectual disability (Wise et al., 2020). The study reported lower ability to independently complete ADLs for those of lower intellectual functioning, but no evidence of age-related changes in ADLs.

To date, studies have mostly involved younger samples (mean age < 50) with co-occurring intellectual disability. Whilst such studies provide important insights into daily living for autistic adults, it is also important to understand the unique challenges and support needs of those who are aging without co-occurring intellectual disability. Those without intellectual disability may be at high risk of neglect and inadequate support provision owing to the apparent ‘invisible’ nature of their autism (Hwang et al., 2017).

Qualitative studies and commentaries involving middle-age and older adults without intellectual disability highlight the need to explore their lifelong support needs and provide preliminary insights into areas in need of support, such as managing co-occurring medical and mental health conditions, and improving public awareness (Edelson et al., 2021; Hwang et al., 2017). They also point to the need to consider environmental and societal factors that may create barriers to their daily living, rather than exclusive focus on individual factors (Edelson et al., 2021; Hwang et al., 2017). Studies involving those with intellectual disability have explored ADLs, pertaining to the performance of basic daily activities. For autistic adults without intellectual disability, it may be more useful to explore performance of IADLs, which are more complex skills involved in independent maintenance of life in and out of the home.

**Fig. 1:**
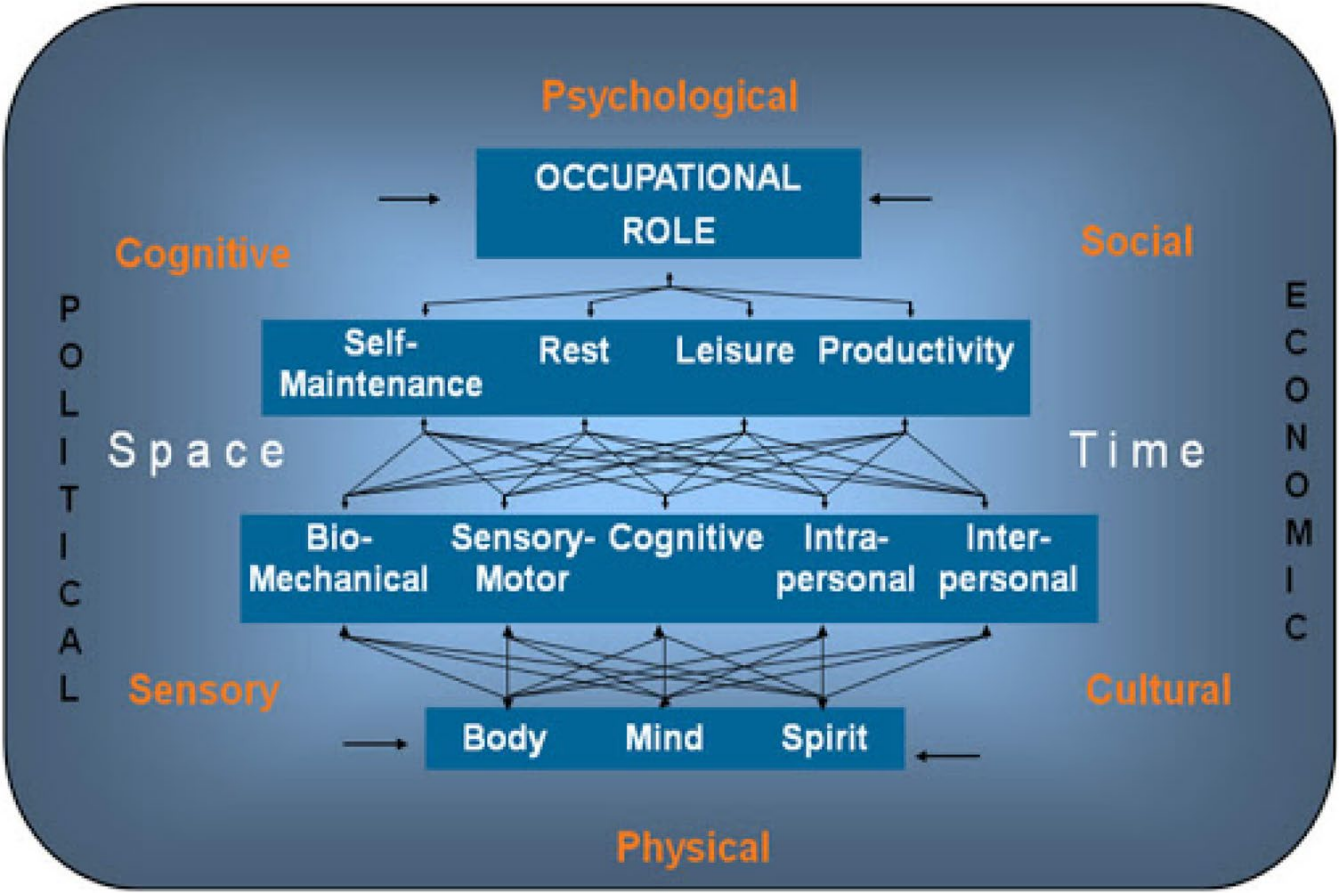
The occupational performance model Australia. Reproduced with permission from “Occupational Performance Model Australia” by Ranka and Chapparo (2011)

There is a paucity of evidence-based, socio-ecological, age-appropriate frameworks for understanding daily living skills in older autistic adults. In this study, we adopted the Occupational Performance Model Australia (OPM(A)) (Fig. 1) (Chapparo & Ranka, 1997; Ranka & Chapparo, 2011) as a potentially useful way to understand performance in daily living skills in autistic adults as they age, and the development of relevant and effective supports for this group. Occupational performance is defined as “the ability to perceive, desire, recall, plan and carry out roles, routines, tasks and subtasks for the purpose of self-maintenance, productivity, leisure and rest in response to demands of the internal and/or external environment” (Ranka & Chapparo, 1997, p. 1) The model identifies occupational performance components that are the physical, psychological and social aspects that determine whether and how well an occupation can be performed. Underlying these components are core elements of body, mind and spirit, the interconnected nature of which interact to provide key underlying resources to achieve occupational performance. The model further contextualises occupations as being supported/hindered by surrounding physical, sensory, cognitive, psychological, cultural and social environments. Finally, the model considers *space* and *time,* both that which is physical (i.e., the objective passing of time or material location of an item) and felt (i.e., the subjective experience of activities and objects) as important factors overlying the entire experience of occupational performance.

Exploring occupational performance using the OPM(A) involves the consideration of three factors: (1) how well an individual can carry out an activity in a real-world context, (2) identifying the person’s capacity/strategy application to tasks and possible reasons for difficulties that are encountered, and (3) the influence of multifaceted contexts on their performance.

The OPMA has been used to guide occupational performance curricula in Australian universities and in practice guides for occupational therapists (Joosten, 2015; Rodger & Kenedy-Behr, 2019). In research, it has been applied as a framework to understand factors that affect occupational performance in populations for whom a diverse range of person and environmental level factors may be influential, such as those who use powered wheelchairs, and obese children (Gill, 2011; Hardy, 2004). Its use enables exploratory research to guide further research and practice. Utilizing the OPM(A) to explore performance of daily activities in aging autistic adults has the potential to provide key insights into their specific challenges and strengths in daily activities. This can in turn lead to a better understanding of how to support autistic adults to lead autonomous and meaningful lives. Here, we adopted the OPM(A) as a framework for understanding the different areas that are involved in and affect the experience of daily activities in older autistic adults without co-occurring intellectual disability.

## Methods

The present study is part of a larger qualitative study designed to understand daily activities in autistic adults, as well as their use of technology in everyday life (see Zheng et al., 2021). In the present analysis, we focus on the questions regarding their experience of IADLs. Ethical approval for the project was granted from the University of New South Wales Human Research Ethics Committee (HC200205).

### Participants

Participants were recruited with the assistance of several relevant community organisations and networks in Australia, who shared study recruitment materials online (Facebook, Twitter, email, internal newsletters). Inclusion criteria included adults aged 50 or older who: had been formally diagnosed with autism spectrum disorder, living in Australia at the time of the interview, with capacity to give their own informed consent, and owned a smart device. Those with intellectual disability were excluded from the current study.

Interested participants were initially asked a series of screening questions to ensure they met eligibility criteria. Participants were excluded if they reported ever having received a diagnosis of moderate or severe/profound intellectual disability. Capacity to consent was also assessed using a quiz designed to assess this in adults with intellectual disability (Horner-Johnson & Bailey, 2013). Eligible participants were then sent the participant information sheet and consent form to read in their own time and asked to re-contact the research team if they wished to participate. Verbal consent was obtained from participants prior to commencing the interview and details of the date and method of consent were recorded in the participant database.

### Interviews

Semi-structured interviews were offered via phone call, video call (Skype or Zoom) or email. To begin, participants were asked to describe their typical day. Next, we asked a series of open-ended questions about the following seven IADLs: transportation, shopping, preparing meals, house cleaning and home maintenance, managing medications, communications, managing finances. Participants were asked about their experiences with these activities in their everyday lives, any challenges they faced, and to discuss any strategies used to manage these activities (see Supplementary Materials for the full interview schedule). Video and phone interviews were audio-recorded and transcribed by the interviewer. A brief (5-min), online demographic questionnaire was emailed to participants at the end of the interview, which contained questions regarding age, gender, postcode, source of income, ethnicity, Indigenous status and housing situation. The Index of Relative Socioeconomic Disadvantage (IRSD) (Australian Bureau of Statistics, 2014) was generated from participants’ postcodes. Participants were emailed electronic gift cards (20AUD per hour) at interview completion.

### Analysis

Analysis occurred in two stages. Stage 1 was deductive, such that the whole dataset was coded into categories using the OPM(A) framework. Responses were coded into which element of the OPM(A) they appeared to fit into at a semantic level: *occupational performance areas, occupational performance components, core elements of occupational performance, environment, space and time.* Some responses were relevant to multiple elements, and not all elements were relevant to participants’ responses. Stage 2 involved looking within each of the coded categories from Stage 1 and conducting an inductive thematic analysis (Braun & Clarke, 2006; Braun et al., 2020) within each of these categories. This approach allowed us to explore common themes across participants’ responses within each category. All coding at both stages was conducted at the semantic level, such that no further interpretation of responses was made. Overall, this approach was deemed most suitable to explore the key experiences of this group with regard to their daily living skills, within the OPM(A) framework. Transcripts were first read by two researchers (YIJH and LZ) to grasp the entirety of the data. One researcher (YIJH) then conducted the analysis. A sample (n = 5) were then sent to the second researcher LZ, who used an empty coding framework (the OPM(A) framework) to code responses. Inter-rater reliability was strong, at 95% agreement and a moderate Kappa coefficient of 0.71. The raters came to agreement regarding some inconsistencies in rating, discussed whether the categories and themes were reflective of the entire dataset, and the final coding framework was established. This method reflects a ‘small q’ approach to thematic analysis by focusing on enhanced reliability, and allocating data to predetermined themes (Braun & Clarke, 2021).

### Community Involvement

This research was conducted with the involvement of an autistic adult. This autistic adult, who is also an academic researcher, was a co-investigator for the grant application that funded this work. They were also involved throughout the research process including study design, development of study materials, recruitment and interpretation of findings, and are a co-author on this manuscript.

## Results

Fifteen participants, aged between 50 and 73 years (M = 60.1, SD = 7.4), participated in interviews over a six-month period (May 2020–October 2020). All participants completed interviews via video call. Demographic characteristics are presented in Table 1. Participants were sampled from six states and territories across Australia, with slightly more female (n = 8, 53%) than male participants. The majority identified as Caucasian (n = 12, 80%). No participants were of Aboriginal or Torres Strait Islander origin.

**Table 1:** Demographic characteristics of interview participants (N = 15)

	n (%)
Age M (SD; range)	60.1 (7.4; 50–73)
Gender	
Male	6 (40)
Female	8 (53)
Non-binary	1 (7)
Location	
Queensland	4 (27)
Australian Capital Territory	3 (20)
New South Wales	3 (20)
Western Australia	2 (13)
South Australia	2 (13)
Victoria	1 (7)
Index of Relative Socioeconomic Disadvantage	
1—most disadvantaged	1 (7)
2	4 (27)
3	4 (27)
4	2 (13)
5—least disadvantaged	4 (27)
Diagnosis	
ASD	8 (53)
Asperger’s syndrome	7 (47)
Income source	
Government pension	10 (67)
Wages/salary	3 (20)
Mixed/none of these	2 (13)
Ethnicity	
Caucasian	12 (80)
Mixed/other	3 (20)
Accommodation type	
Own house/renting	13 (87)
Public or community housing	2 (13)
Household	
Living alone	5 (33)
Living with partner and/or family	8 (53)
Living with housemate	2 (13)

### Occupational Performance Areas

There are four occupational performance areas described within the OPM(A); rest, self-maintenance, productivity and leisure/play occupations. The authors note that “the classification of occupations into these categories is an idiosyncratic process” (Ranka & Chapparo, 2014). In our study, all seven activities were described by participants as relating primarily to the self-maintenance area. That is, they were completed for the purposes of preserving health and wellbeing and participants did not tend to associate these activities with rest, productivity or leisure.

### Components of Occupational Performance

The components of occupational performance describe the underlying skills required to execute a task and include biomechanical, cognitive, sensory-motor and psychosocial (intrapersonal and interpersonal). Participants’ experiences in daily living skills are described below through these components.

#### Biomechanical

Biomechanical components are the operation and interaction of the physical structure of the body during a task (Ranka & Chapparo, 2014). Participants reported varying levels of biomechanical aptitude in performing their daily activities. Some expressed no difficulty in their physical ability to complete housework, driving and other daily tasks.

I can do all [house maintenance]. I can especially do gardening. And the gardening includes things like constructing trellis along the top of my fence and those kinds of physical things as well and construction things. [P01]

Conversely, some participants described that the existence of varied, specific physical health conditions restricted their ability to complete physical tasks.

I can't stand for any length of time. Mainly related to chronic fatigue because I haven't got the energy to support my own weight. My partner does most of the evening meals. [P08]

I've got hyper mobile Ehlers-Danlos syndrome which means that I can hurt myself very easily, from doing not much… Things like cleaning kitchen benches like you know, physically that just really hurts my fingers. [P10]

#### Cognitive

Cognitive components are the mental process related to performing a task and may include attention, memory, planning and problem solving (Ranka & Chapparo, 2014). Participants commonly reported executive challenges in memory and attention. These often complicated the completion of tasks for which they are otherwise physically or intellectually capable.

Housework kind of, again, it gets overwhelmingly complicated and I do forget at times or get distracted. Like ‘oh need to do this cleaning. Oh but I need to do this’, and ‘that’s more important like an appointment so I need to do that first’, Or ‘that’s got a deadline on it so I need to do that’. So I lose track of what I was intending to do…. I’m technically, intellectually and physically capable to do so but become very overwhelmed and distressed when I’m trying to do so. [P06]

A prominent theme within cognitive aspects of daily activities was a preference for familiarity, predictability and routine. As a result, participants often took time to plan and prepare for a variety of daily situations.

I don’t function without structure, so I need the structure which means I need to plan things out and that’s exhausting. It almost feels like whenever I do a task I am relearning or recreating the task step-by-step as I go along. I have to step through each part of it to get it done. And so, yeah, things end up being done slowly. [P05]

A systematic approach or strategy was often developed for task completion, especially shopping.

I start writing it when I start wanting stuff. Usually just a few days before start adding, adding, adding. And I try to write it in the general order of where the aisles are. So then I’ll just go from the top and down, down. And then I should be able to grab everything. Then I check at the end that I’ve got everything on the list. [P03]

#### Sensory Motor

Sensory motor components describe the sensory input (auditory, visual, tactile, gustatory, olfactory, proprioceptive and vestibular) and motor responses of the body during task performance (Ranka & Chapparo, 2014). Participants often described how sensory experiences created challenges for task completion. Sensory experiences were reported to sometimes override the person’s ability to perform biomechanical and cognitive components of the tasks.

I have a number of sensory issues that vary in their intensity. I'm hypersensitive to water and certain touch, so there are times where I can't get my hands wet or moist and that has quite a profound impact on my ability to cook. [P02]

When I’m around perfume, what little brain capacity I have heads off and disappears, so I get almost unable to talk, not unable to talk but unable to talk sense. [P01]

#### Intra-personal

Intra-personal components refer to internal psychological processes used during task performance (including motivation, satisfaction, self-esteem and self-confidence) (Ranka & Chapparo, 2014). Participants expressed they had over time learnt to use self-motivation and willpower to complete daily activities that they found uncomfortable but necessary, such as making phone calls or going shopping.

I just can't face going to buy anything on [shopping] list very often. And then one day I'll just wake up and go OK. I can do it today and I will go to a shopping centre [P10]

I kind of resort to doing the things I have to do, and that’s just done by brute force, sheer force of will. Just making myself do stuff. [P05]

When we first got married I wouldn’t even get on the phone. I hated it, absolutely dreaded it. So over time, I still have moments when the phone rings and I’m like ‘aahh’. Generally speaking I’m a lot better now. I’ve gotten used to the fact that I’ve got to get on the phone. [P07]

#### Interpersonal

Interpersonal components are the continuing and changing interactions between a person and others during task performance (Ranka & Chapparo, 2014). Participants who had partners (n = 7, 47%) often shared their daily tasks with their partners, especially those they found challenging. Partners often reminded autistic adults to complete certain tasks and provided social support.

I have relinquished most household responsibilities over the last number of years. So I used to do all of those [housekeeping] and now I don't. Yes. I ask [partner]. He's very, very good. He's excellent. And quite happy to do all of those things. And I'm very unhappy to do them. So we've come to an agreement.” [P10]

“Say when it comes to diet, to eat the right appropriate foods. I’ll go, ‘I’ve just had [fatty food]’ and [partner] will say, ‘I told you you shouldn’t have that, it’s too fatty’. I forget things. I shouldn’t have had too much of that because it’s too fatty. Because I’ve had heart problems so I’ve got to watch what I eat. [P07]

Several participants also received help from support workers or health professionals. Whilst helpful, participants expressed difficulty in negotiating their specific and varying needs with support workers.

The deal I have with [the support worker] is each week I will identify what needs to be done that week, where the priorities are, and I will indicate that. A lot of the workers want to come in and do it their way. Do what they want their way and go. And that doesn’t work that way because some things are more important than others. And I had to have deals with providers that they would go along with that but they don’t like it all that much. [P01]

### Environments

The external environment in which occupational performance occurs can shape the nature of the performance of an activity. Aspects of the environment which influenced the occupational performance of our autistic participants included cognitive, sensory, social, cultural and economic.

#### Sensory Aspects

Sensory aspects of the environment describe the sensory surroundings of a person (Ranka & Chapparo, 2014). They often created challenges for autistic adults in performing their daily activities.

In the supermarket, the lights are just so bright, it’s like, it makes me feel sick. And my eyes… I just wear a hat and you know you can’t really wear sunglasses in the shop because you can’t see things properly that you want to buy. Sometimes I wear earplugs because they just suddenly start saying these things on the speaker. Like you’ve got a nice song then all of a sudden they start having an announcement it I just jump…. and if I go to a normal checkout and they swipe each thing beeps so quite often I have to ask the lady to turn the beeper down because it makes me jump. And if you’re getting like 50 items I don’t want 50 beeps. [P03]

#### Cognitive Aspects

Cognitive aspects of the environment refer to the complexity of the context and the ease with which it can be interpreted (Ranka & Chapparo, 2014). Participants expressed a preference for familiar places. Places and environments that were unfamiliar or unpredictable were often described as unsettling and contribute to feelings of anxiety, stress or unsafety.

When I was in [old job] I had to do a lot of travelling running around to jobs. That was quite stressful at times, especially if I didn’t know the place. I’ve had plenty of panic attacks on that sort of area. To get to this place on time and all that sort of thing and to find, to get the map and think ‘oh my gosh’. [P07]

I don't feel safe if I'm in unfamiliar surroundings, or if there's unfamiliar people around me. So anything unfamiliar, I feel unsafe with. So I do everything I can do to go out in ways that are safe for me. I prefer to be home in my unit where there is a sense of familiarity with all of the objects and things in my house are where they should be. And I don't like people coming into my space unless I have invited them and I have sufficient notice. [P02]

#### Social Aspects

Social aspects of the environment refers to an organized structure created by the patterns of relationship between people who function in a group (Ranka & Chapparo, 2014). Participants in this study reported a general preference for solitude, but not isolation, in daily living. Participants were aware that their methods, needs and preferences for social interaction were different to neurotypical individuals. As a result, they had developed and negotiated the methods and frequency with which they interact with others such as family and friends.

I like my own time. I like being able to choose what I am going to do. And that might involve occasionally catching up with someone for a coffee or it might include talking to you [the interviewer], but I manage those things. I won't have more than one of them in a day if I can help it because otherwise, I will just feel drained from the interaction with people. [P02]

I'm one of those autistic people that really need a focal point to communicate on. If we're going to go to say, the art gallery, I would be focused on the exhibition and then maybe afterwards talking about or talking about the work as we're going. But I can't handle these people who go there going to do something and they just talk about nothing. Chit chat. Because I'd rather be there alone. And because I'm there for a purpose, so it's very difficult. It's very difficult to talk to people if there's no focal point. [P08]

Moderated, indirect forms of communication (i.e., text, email, messenger apps) were one way that allowed the person to process and respond more comfortably, as opposed to direct methods such as phone calls that were described as stressful and anxiety-provoking due to a sense of immediacy. However, face-to-face communication was preferred in some settings as it allowed more visual cues to help understand what was being communicated.

I don't like using phones. I don't like using face-to-face things because they imply a sense of immediacy. My phone rings and I've got to answer it, whereas if I get a text or something like that in messenger I can reply when I'm ready to, rather than the person who is contacting me effectively imposing their timetable on me. [P02]

I have to be in the right frame of mind to make a phone call. I'm quite capable of making a phone call and I know some people do find it really difficult. I can. Though I need to have pen and paper handy, or have some notes made. So that I know what I'm going to talk about, like I have to think about what I'm going to talk about. I can't just wing it? And I hate getting phone calls out of the blue and like just random phone calls [P10]

#### Cultural Aspects

Cultural aspects of an environment refer to an organized structure of systems, values, beliefs and customs (Ranka & Chapparo, 2014). Cultural barriers stemmed from a lack of understanding and/or negative attitudes regarding autism.

I think now that autism, my disclosure that I have diagnosed autism is upsetting and irritating some people. And I have come to the view that they are treating me as if they’ve heard stories that convince them… that I’m mad or bad. That I’ve got a mental illness that they can’t cope with or that I’ve done something terrible. [P01]

#### Economic Aspects

Economic aspects refer to explicit and implicit costs and financial systems that influence a person (Ranka & Chapparo, 2014). Participants commonly expressed that their finances were inadequate for their sensory and physical support needs as well as their hobbies.

The biggest problem that I have at the moment is that I don't have enough money... In order to build the house, because of my thermos-regulatory disorder, I need all sorts of insulation in the walls try and keep the house at a regular temperature year round so that there aren't sort of severe fluctuations in temperature inside the house. [P07]

I need to order a [groceries] delivery [because of physical disability], which is a terrible waste of money because it’s $13 in delivery cost. [P10]

Participants were frugal and conscious spenders due to limited income.

I'm a sort of broke pensioner I have no sort of assets I just get a disability pension each fortnight. I do a few little odd jobs here in there I earn a little bit of money for. But it's difficult on a disability pension to actually earn much more money [P12]

## Discussion

This study contributes important knowledge regarding experiences of daily activities for middle-aged and older adults on the autism spectrum without co-occurring intellectual disability. Whilst we lack population-based data regarding the demographic characteristics of autistic adults in Australia, the sample’s gender and ethnicity closely resembled that of the cohort that participated in the Australian Longitudinal Study of Autism in Adulthood (ALSAA) (Arnold et al., 2019). The specific nature and extent of challenges experienced in daily activities varied for each participant. Notwithstanding, this study found that the occupational performance of daily activities for middle aged and older autistic adults was influenced by (i) person-level factors such as the ability and characteristics of the adult, (ii) the environment within which activities are being performed, and (iii) issues arising at the intersection of the adult and their environment (i.e., person-environment fit).

**Fig. 2:**
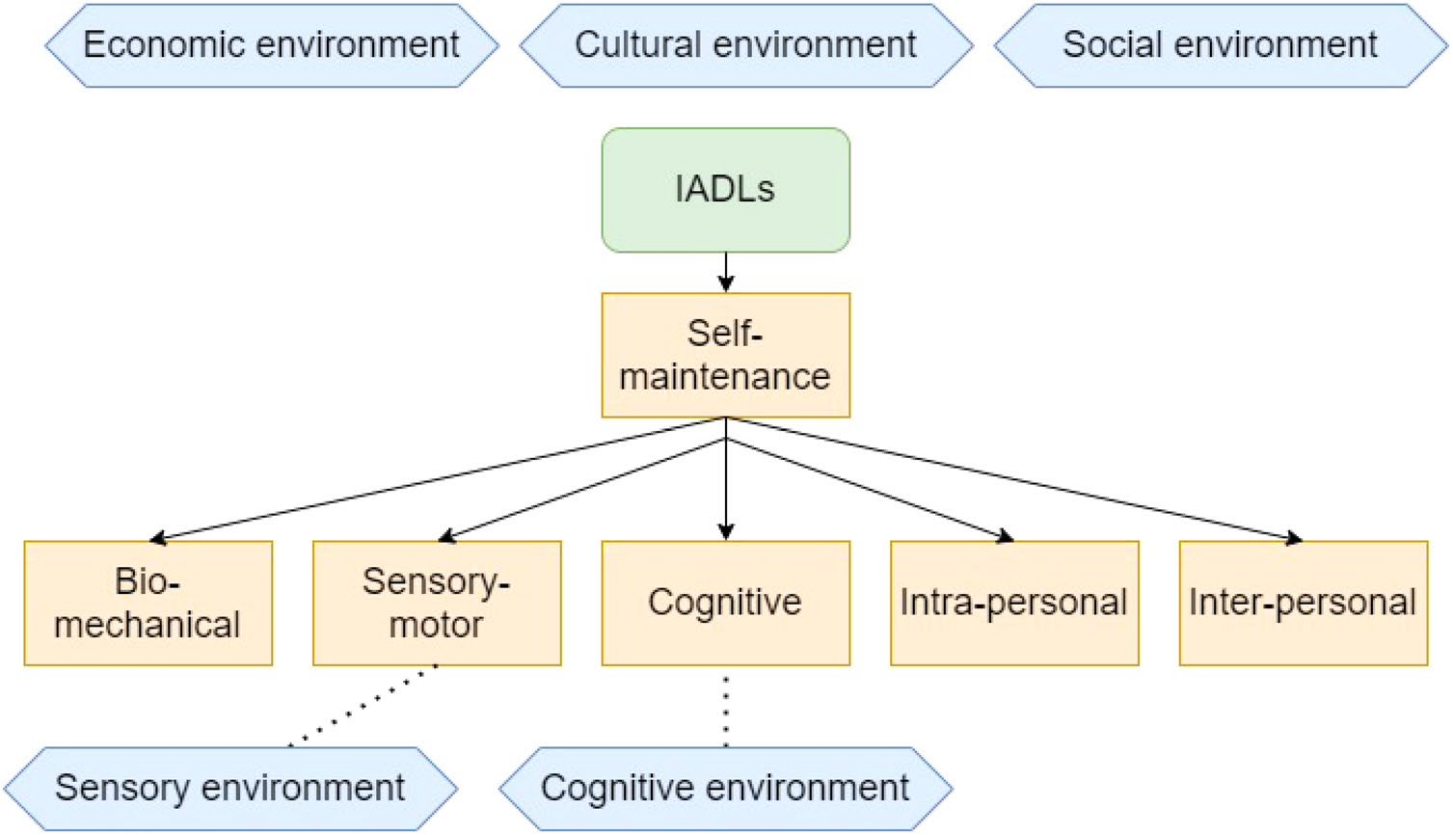
OPM(A) elements relevant to instrumental activities of daily living for older autistic adults

The refined OPM(A) model resulting from this work (Fig. 2) presents a useful framework for support workers and other professionals to guide the design and delivery of appropriate supports. It highlights priority areas that impact autistic adults’ daily activities, how they may be affected by person-environment fit as well as the broader contexts within which daily activities occur. According to the present findings, IADLs, which are primarily undertaken for the function of self-maintenance, operate through, and are affected by, a range of person factors and their interaction with the environment. For the person, their bio-mechanical ability, sensory-motor aspects, cognitive differences, intra-personal processes and interpersonal support all affected their activities. At the environmental level, IADLs were affected by cognitive, social, sensory, cultural and economic contexts.

### Person-Level Factors

Person-level factors affecting occupational performance included performance of biomechanical, cognitive, sensory-motor and intrapersonal components of the task. Biomechanical challenges were varied in nature and related to co-occurring physical conditions. This finding is consistent with the high rates of co-occurring medical conditions in autistic adults of all ages (Sonido et al., 2020). Sensory-motor components of daily activities were also strongly featured in participants’ responses, with participants reporting varying and often multiple sensory sensitivities across olfactory, vision, gustatory, tactile and auditory sense. These made it difficult to engage in activities both in and outside the home. Cognitive components of tasks were reported as challenging by participants and converged on issues related to executive functioning. Most common were maintaining attention and staying focused to task completion, memory and habit formation, and managing time. Participants discussed difficulties in planning and executing tasks highlighting that challenges in task completion for older autistic adults may be less related to having the physical capacity to do a task, but more about the specific cognitive requirements of a task in relation to sequencing and completing a number of consecutive steps. Physical comorbidities and sensory issues were often reported to compound these cognitive issues.

While declines in physical and executive function are also apparent in older age for neurotypical adults, these findings evidence that for those on the spectrum, there is a need for support in both physical and cognitive aspects of daily activities from middle adulthood (50 +) with a personalised approach that considers the varying needs of the individual. In addition, it will be important for supports and services to consider how they may support the autistic adults to navigate the sensory components of a task to improve overall occupational performance.

Participants also expressed having learnt over time to use intrapersonal processes of motivation and resilience to cope with challenges faced in daily activities, particularly in navigating sensory components of tasks, as well as dealing with social or unfamiliar environments that were stressful or anxiety-provoking. While some participants had developed specific strategies to perform daily activities, others simply accepted such discomforts as inevitable and learnt to forcibly endure them. This is problematic given research indicating a tendency for autistic adults to use maladaptive strategies in self-regulation of internal states, ‘masking’ of their autistic traits, and the flow-on effect of this onto negative psychological wellbeing (Cage et al., 2018; Cai et al., 2018). There is a need for service providers and health professionals to work with autistic adults to devise productive and adaptive strategies to deal with challenges in daily activities.

### Person-Environment Interaction

Support workers were reported to assist with daily activities, though it was common for participants to report difficulty negotiating their specific needs with them. Partners were the preferred source of support and an important enabling factor in performing daily activities. This is aligned with studies involving non-autistic adults that also find informal support from family and friends to be important as adults age (Abdi et al., 2020). In autistic adults, partners may have been preferred sources of support due to the preference for familiarity and solitude that may be interrupted by a support worker. A partner with whom the autistic adult is well acquainted may cause less intrusion and more harmony with their usual environment. It will be important to consider a central role for partners in development of supports for autistic adults, and simultaneously to consider the support needs and health of the partners themselves who may also be aging. If desired, support for autistic adults to establish and maintain such relationships may be beneficial to improving daily activities.

In the community, sensory and cognitive components of the environment created challenges in performing daily tasks. For example, public places often triggered sensory discomfort and sensitivity. Also, there was particular anxiety and distress associated with situations and environments that were unpredictable or unfamiliar. Together, such challenges created an additional layer of complexity for autistic adults in choosing and planning for daily activities outside the home. These findings provide evidence that significant challenges in performing cognitive and sensory aspects of tasks are apparent in middle and older adulthood. Preferences for predictability, familiarity and routine observed in this study closely reflect ‘intolerance of uncertainty’, a dispositional factor found to be elevated in autistic people of all ages (Hwang et al., 2019; Jenkinson et al., 2020). Given that intolerance of uncertainty and sensory sensitivities are found to be intercorrelated and associated with clinically-significant levels of anxiety in autistic adults (Hwang et al., 2019), better supporting these aspects of daily living for this population may have significant effects on decreasing anxiety.

Whilst aforementioned findings suggest autistic adults often developed person-level strategies to cope with challenges in daily activities, it is apparent that several aspects of the autistic individuals’ lives are made inaccessible by aspects of the society in which they live, reflecting a social model of disability (Oliver, 2013). In addition to supporting adults to manage their challenges, it is equally important for a consideration of the disabling aspects of the environment that may be dealt with to reduce the onus on the adult to cope with these challenges. In addition to consideration of sensory triggers in the community, efforts should include “adjustments to enhance environmental predictability and the autistic individual’s sense of control, while allowing for the uncertainty and flexibility necessary in educational, work, and community environments” (Lai et al., 2020, p. 435).

### Environmental Factors

Finally, performance of daily activities was impacted by broader economic, social and cultural aspects of the environment. Participants expressed feeling restricted with their finances in general, and that this affected their ability to access required supports and engage in leisure activities. Most participants were receiving income in the form of government pensions. The lack of available finances may be exacerbated for autistic adults due to low rates of employment (Hedley et al., 2017) and high rates of physical and mental co-occurring conditions, which may increase support needs but decrease employment opportunities. There is a need to increase opportunities for sustained employment for autistic adults throughout adulthood, thereby increasing financial freedom in later life, as well as to develop affordable supports that are accessible to autistic adults in older age.

Participants expressed specific preferences regarding social contact and communication in their day-to-day lives. There was a tendency to prefer solitude, though not isolation, in that certain social interactions were appreciated and welcome. This was determined by the other party’s apparent awareness and respect for the autistic adult’s preferences and alignment in interests or objectives of the social encounter between parties. More indirect, mediated forms of communication were also preferred, which allowed our autistic participants to process and respond in a controlled, comfortable pace and method. An exception to this was where face-to-face contact would be useful for gaining visual cues to better understand what is being communicated. These provide important considerations for support workers, health professionals and others in supporting and interacting sensitively with autistic people in a comfortable and effective manner.

Unfortunately, it was common for participants to speak of cultural barriers in awareness and acceptance of autism. Resistance, ignorance and a lack of formal and informal support was experienced from peers, professionals and those from the general public. It is important for both formal recognition and informal awareness of the sensory, cognitive, social and cultural needs that autistic adults have in daily life especially in public, to better facilitate performance in daily activities for this population.

Cultural factors may have contributed to the difficulties with support workers reported by the sample. This may be reflective of a lack of understanding and acceptance of the unique nature of support required for autistic people. Sensory needs or the need for familiarity and routine are not formally recognised as a health or medical condition. As a result, they may be misconstrued as the autistic individual being picky, sensitive or demanding. In addition, those on the spectrum will need additional time and support to complete daily activities such as shopping. There is therefore a need for increased respect and patience on behalf of others with whom autistic adults may interact with throughout their day. Such acknowledgment should be the focus of large-scale initiatives to increase public awareness or more targeted efforts for educated workforces in shopping centres, post offices and other public locations. Recent efforts in Australia have seen the introduction of “quiet hour” or “low sensory shopping” across certain grocery stores. Such efforts might be particularly beneficial for autistic adults in light of the present findings, although future efforts, including in other public areas, should focus on universal design to allow for inclusive environments for all.

### Limitations and Future Work

This study focused specifically on the experiences of those without co-occurring intellectual disability. People with intellectual disability represent a substantial proportion of the autistic population (33%) (Redfield et al., 2020) and it is likely they will have unique needs and challenges that must be understood and addressed via future studies. Also, due to the method of recruitment being primarily via online methods, our sample may reflect adults who are skilled in technology use and less able to represent those who lack access to the internet due to location, socioeconomic status, as well as those who are older. This may have in part explained the relatively ‘young’ sample. Our sample is younger in age than what is traditionally considered in other studies of aging (65 +). Notwithstanding this, the younger inclusion criteria set for the study enabled us to explore daily experiences of middle-aged adults also, for whom such knowledge is lacking.

This study relied on the self-report of autism diagnosis in participants. Whilst not clinically validated, the likelihood of false responses was minimised by asking participants a series of screening questions regarding their diagnosis upon expressing interest to the study, including “have you been formally diagnosed with autism”, “What is the name/label of your diagnosis”, “What year were you diagnosed” and “who was the professional who provided your diagnosis”. Given the relatively small reimbursement for up to two hours of in-depth questions relating to autism it is unlikely that false responses were present. Similarly, exclusion of those with intellectual disability was based on participants’ self-report. Whilst this may be a limited approach, it is the experienced opinion of the research team that successful completion of the extended interview and its complex questions required intellectual ability that aligned with this criteria. These methods also allowed participation of older autistic adults without the additional burden of copying and emailing diagnostic reports, which may have created a significant barrier to participation for this hard-to-reach population.

In this study, participants reported varying strategies to manage their daily activities that were unique to their specific challenges and preferences. These included a range of psychological strategies, physical supports (e.g., noise cancelling headphones), and planning. To appropriately support older autistic adults, it will be important to understand these strategies and work with the adult to further develop supports and services that are tailored, adaptive and sustainable.

While this study fills a gap in our understanding of daily activities and need for support in middle-aged and older autistic adults, there remains little qualitative evidence of the challenges in daily activities faced by younger adults also. Younger adults are more likely to be involved in employment and education activities, entailing unique difficulties in negotiating social and communication preferences such as the preference for solitude. Also, strategies to manage any challenges in daily activities that were apparent in the present sample may be different or less developed in younger cohorts. Resultantly, the nature and target of supports needed will likely differ with age.

## Conclusion

This study presents the first qualitative exploration of daily activities for middle-aged and older adults on the autism spectrum. Aspects known to be elevated or unique in autistic individuals, such as sensory sensitivities, intolerance of uncertainty, medical conditions and socio-communication preferences, impact daily functioning in mid- to later life. Heterogeneity in participants’ specific experiences and needs attest to the need for individualised supports in these areas, delivered by someone who understands and respects the adults’ varying needs. There are financial costs associated with enabling autonomy and comfort in daily activities, which can cause additional difficulties for autistic adults who find themselves financially strained. A focus on person-environment fit is needed, as many challenges are further exacerbated by non-compatible environments. Adults should also be supported to develop effective ways of managing challenges that may be inevitably faced in the environment.

## Supplementary Information

Below is the link to the electronic supplementary material.

Supplementary file1 (DOCX 32 kb)
