# Supplementary material for: Experiences of Performing Daily Activities in Middle-Aged and Older Autistic Adults: A Qualitative Study
Source: J Autism Dev Disord. 2022 Feb 25;53(5):2037–49. doi: 10.1007/s10803-022-05473-7 (PMC10123035; doi:10.1007/s10803-022-05473-7)
Supplement: Supplementary file 1 — Supplementary file1 (DOCX 32 kb) [file 10803_2022_5473_MOESM1_ESM.docx]

**Interview Schedule**

**[Start Recording]**

1. Can you tell me a bit about yourself (e.g. what you do for work or study, tell me about your family, where you live etc).
2. Can you briefly tell me what a typical day looks like for you (e.g. from when you wake up in the morning to when you go to bed).

**[ Show Icon Board]**

1. Now I’m going to ask you about some activities you may encounter in your daily life. You can see them on this board here. We’re going to start with this one [point to the ‘transportation’ icon].
2. [Transportation]

- In general in your daily life, do you face any problems getting around, or getting to and from places?

*If yes:*

1. *What kind of problems?*
2. *How do you currently manage these problems?*
3. Next we are going to talk about this one [Point to ‘Shopping’ icon]

- In general in your daily life, do you face any problems shopping for the things you need (e.g. groceries, clothes, electronics?)

*If yes:*

1. *What kind of problems?*
2. *How do you currently manage these problems?*
3. Next we are going to talk about this one [Point to ‘Preparing meals’ icon]

- In general in your daily life, do you face any problems preparing food for yourself?

*If yes:*

1. *What kind of problems?*
2. *How do you currently manage these problems?*
3. Next we are going to talk about this one [Point to ‘Housecleaning and home maintenance’ icon]

- In general in your daily life, do you face any problems looking after your home? (e.g. doing laundry, washing dishes, maintaining the garden etc)

*If yes:*

1. *What kind of problems?*
2. *How do you currently manage these problems?*
3. Next we are going to talk about this one [Point to ‘Managing medications’ icon]

*Ask: Do you currently take any medications? If yes, continue asking the following questions. If no, move onto ‘communication’ section.*

- In general in your daily life, do you face any problems managing any medications you take? *If yes:*

1. *What kind of problems?*
2. *How do you currently manage these problems?*

**[Break: “We’re almost halfway through. Do you want to take a break?” If yes, take a 5 minute break. If no, keep going]**

1. Next we are going to talk about this one [Point to ‘Communications’ icon]

- In general in your daily life, do you face any problems getting in contact with other people (e.g. getting in touch with family, friends, service providers etc)

*If yes:*

1. *What kind of problems?*
2. *How do you currently manage these problems?*
3. Next we are going to talk about this one [Point to ‘Managing finances’ icon]

- In general in your daily life, do you face any problems managing your money?

*If yes:*

1. *What kind of problems?*
2. *How do you currently manage these problems?*

**Demographic Questionnaire**

1. What is your date of birth?
2. What was your assigned sex at birth?

- Male
- Female
- Intersex/indeterminate/unknown

1. What is your current gender identity?

- Male
- Female
- Trans male/Trans man
- Trans female/Trans woman
- Genderqueer/Gender non-conforming
- Different identity (please state): _________________________________________

1. What is your postcode?
2. What is your main source of income?

- Wages/salary
- Government pension or allowance
- Child support or maintenance
- Superannuation or annuity
- Workers Compensation
- No/None of these
- Any other regular source, please specify _____________________________________

1. Do you have any diagnoses other than Autism? Please list all that apply.
   e.g. Intellectual disability, Down syndrome, Anxiety, Depression etc
2. What is your ethnic background?

- Caucasian
- Asian
- Hispanic
- African
- Middle Eastern
- African American
- Other, please specify: _______________________________________

1. Are you of Aboriginal and/or Torres Strait Islander origin?

- No
- Aboriginal,
- Torres Strait Islander
- Aboriginal and Torres Strait Islander

1. What kind of accommodation do you currently live in?

e.g. privately rented flat or house, residential or nursing home, supported lodging etc

1. Please list all those who share your residence with you (number of people and relationship to you) e.g. partner, relatives, housemates etc

| Person | Relationship |
| --- | --- |
| 1 |  |
| 2 |  |
| 3 |  |
| 4 |  |
| 5 |  |
| 6 |  |
| 7 |  |
